# PATIENT PERCEPTIONS OF A PERSONALIZED MUSIC PLAYLIST PROGRAM FOR HOSPITALIZED OLDER ADULTS

**DOI:** 10.1093/geroni/igae098.2468

**Published:** 2024-12-31

**Authors:** Kayla Wong, Maanasa Nandigam, Victor Nguyen, Angela Morka, Eliza Kiwak, Judy Li, Jody Sharninghausen, Richard Marottoli

**Affiliations:** Yale University, New Haven, Connecticut, United States; Yale University, New Haven, Connecticut, United States; Yale University, New Haven, Connecticut, United States; Yale University, New Haven, Connecticut, United States; Yale University School of Medicine, New Haven, Connecticut, United States; Yale University School of Medicine, New Haven, Connecticut, United States; Yale University School of Medicine, New Haven, Connecticut, United States; Yale University School of Medicine, New Haven, Connecticut, United States

## Abstract

Personalized music playlists may be therapeutic for hospitalized older adults. As part of the “Creativity in Aging” project at Yale Geriatrics, this study aims to bring personalized music to hospitalized older adults and explore their perceptions of its effect on emotional and physical health. Patients (N = 57) hospitalized in an inpatient geriatrics unit participated in the program from October 2023 to February 2024. Qualitative data were collected from 19 patients. Trained undergraduate volunteers took detailed “music histories” from patients and, when appropriate, their family caregivers (e.g. patients with dementia), and created personalized playlists using iPads. Patients could listen to their playlists during volunteer shifts throughout their hospital stay. Pre and post-listening surveys were conducted to elicit patient perceptions through brief semi-structured interviews and ratings on a Likert mood scale. A qualitative thematic analysis using the principles of grounded theory was performed to create thematic categories of patients’ perceptions. Two coders open-coded separately then came together to agree on codes and themes. Saturation was reached, as no new themes were generated at the end of the thematic analysis. Five core themes emerged from the analysis: patients expressed feeling 1) calmer and more relaxed, 2) generally more positive, 3) reminiscent, 4) a want to sing and dance, and 5) physically and emotionally healed after listening to music. Constructive feedback included technical issues with iPads and wanting to listen to playlists more often. We aim to increase accessibility and expand the program to older adults in other areas of the hospital. The first two listed authors (MN and KW) contributed equally.

